# Metabolic Syndrome and Inflammation: A Critical Review of *In Vitro* and Clinical Approaches for Benefit Assessment of Plant Food Supplements

**DOI:** 10.1155/2013/782461

**Published:** 2013-02-27

**Authors:** Chiara Di Lorenzo, Mario Dell'Agli, Elisa Colombo, Enrico Sangiovanni, Patrizia Restani

**Affiliations:** Dipartimento di Scienze Farmacologiche e Biomolecolari, Università degli Studi di Milano, Via Balzaretti 9, 20133 Milan, Italy

## Abstract

Metabolic syndrome is defined as the clustering in an individual of several metabolic abnormalities associated with insulin resistance, type 2 diabetes, and obesity, in which low-grade chronic inflammatory activity is commonly observed. Part of the European Project PlantLIBRA is concerned with methods to assess the benefits of plant food supplements (PFSs) in countering inflammatory activity and metabolic syndrome. This paper summarizes the current methods used for benefit assessment of PFS, taking into consideration only *in vitro*, in silico, and clinical methodologies used to investigate the anti-inflammatory properties of plants. No in silico studies (using computer simulation) related to metabolic syndrome were found; these methods appear to be used exclusively for identifying or testing potentially effective compounds in drug development. Most *in vitro* methods for the assessment of beneficial effects of botanicals or plant food supplements in diabetes were based on a quantitative polymerase chain reaction (PCR), whereas the preferred kind of clinical study was the double-blind randomized controlled clinical trial. Only two parameters were observed to change after treatment with botanicals in both *in vitro* and *in vivo* studies: interleukin-6 and tumour necrosis factor-**α**, and these biomarkers should be carefully considered in future studies for PFS benefit assessment.

## 1. Introduction

Metabolic syndrome (MS) defines the clustering in an individual of multiple metabolic abnormalities [1]. World Health Organization and programs including the National Cholesterol Education Program (NCEP) and Adult Treatment Program III (ATP III) have now agreed [2, 3] to consider MS as a disease characterized by five traits: (1) increased abdominal girth, (2) low levels of high-density lipoprotein cholesterol (HDL-C), (3) hypertriglyceridemia, (4) hypertension, and (5) fasting hyperglycemia. A low-grade chronic inflammatory activity is commonly observed in metabolic diseases such as obesity and type 2 diabetes (T2D).

A major shortcoming of current definitions of MS is the lack of inclusion of measures of a proinflammatory state and oxidative stress [4, 5]. The multicenter Insulin Resistance Atherosclerosis Study had shown a linear relation between the inflammatory marker C-reactive protein (CRP) and a number of metabolic disorders [6]. Other proinflammatory markers known to increase in patients with MS include fibrinogen [2], cytokines such as interleukin-6 (IL-6), and tumour necrosis factor-*α* (TNF-*α*).

Type 2 diabetes (T2D) is considered an MS-related disease and an inflammatory disease. As with MS, patients with T2D show higher levels of circulating CRP, fibrinogen, plasminogen activator inhibitor (PAI), and proinflammatory cytokines such as interleukin-1*β* (IL-1*β*) and IL-6. Circulating levels of IL-18 have been reported to be elevated in subjects with the metabolic syndrome and closely associated with the biomarkers of the syndrome to predict cardiovascular events and mortality in populations affected by MS-related diseases [7]. In patients with T2D, metabolic stress promotes insulin resistance and activation of IkB kinase-*β* (IKK*β*) and JUN N-terminal kinase (JNK), which suggests that these kinases have key roles in the pathogenesis of this disease [8]. IKK*β* activates nuclear factor-*κ*B (NF-*κ*B) which induces expression of NF-*κ*B-dependent genes such as proinflammatory cytokines (e.g., TNF*α* and IL-1*β*), so that the suppression of this transcription factor could reduce metabolic disorders and the complications occurring in diabetes (retinopathy, nephropathy, and neuropathy) [8].

In the last 10 years, the link between inflammation and nutrition has become increasingly apparent [8–10]. It has been shown that excessive macronutrient intake can contribute to the inflammatory response occurring in MS [11], whereas some dietary polyphenols are able to reduce the incidence of MS, including diabetes [12]. Many of the metabolites occurring in plants are now recognized as useful for the maintenance of human health, hence the recommended use of plant food supplements (PFSs). A methodology for the safety assessment of botanicals has been promoted by EFSA (European Food Safety Authority), but major bottlenecks remain in its implementation.

The European Project PlantLIBRA (acronym for plant food supplements: levels of intake, benefit, and risk assessments) aims to promote the safe use of PFS or botanicals and the measurement of the risk/benefit ratio related to their consumption. Part of the project is devoted to the discovery of methods for the evaluation of the benefits of PFS and their application and validation. The first step was to review the evidence for PFS benefit in epidemiological, clinical, and intervention studies, in particular the value of PFS as anti-inflammatory agents [13, 14].

The aim of the present paper was to identify the *in vitro* and *in vivo* methods that can detect a decrease of the inflammatory biomarkers that play a key role in metabolic syndrome and diabetes.

## 2. Methods

### 2.1. Source and Search Strategy

The following databases were searched electronically to identify relevant articles published up to September 2011: PubMed/Medline, SciFinder Scholar, and Cochrane Library. Search limits were *in vitro*, *in silico*, *clinical* methodologies, and the European languages, without limits of year of publication.

A search strategy was developed for each electronic database using specific medical subject heading (MeSH) terms (e.g., inflammation mediators, C reactive protein CRP, and metabolic X syndrome) in addition to relevant text keywords (plant extract, plant preparation, methods, and analytical approaches).

The same MeSH terms were used in the T2D area and in the MS area to search for inflammatory biomarkers and plant extracts, and the specific terms diabetes mellitus, noninsulin-dependent OR diabetes mellitus, and type II were combined with relevant keywords (plant extract, plant preparation, methods, and analytical approaches).

Titles and abstracts of retrieved citations were first screened to identify publications reporting *in vivo* methods developed in humans, *in vitro* and *in silico* methods used in inflammation conditions related to MS or diabetes. Animal studies were not considered since PlantLIBRA neither uses nor promotes *in vivo* experiments on animals. Other exclusion criteria were the use of plant ingredients for homeopathy, topical use, aerosol/inhalation, and hygiene products. Reviews, commentaries, and patents were also discarded.

## 3. Results and Discussion

The search by title and abstract retrieved 46 papers for MS and 68 for diabetes. After removal of duplicates and application of the inclusion/exclusion criteria, the total number of papers was 43. Papers were also rejected if they were not in a European language. *In silico* methods for assessing PFS benefit in MS-related diseases were not found. All the studies selected for diabetes were related to T2D.

### 3.1. Metabolic Syndrome Studies

Neither *in silico* nor *in vitro* studies for assessing inflammation in metabolic syndrome were found. Although diabetes is one of the features of MS, the methods related to diabetes have been considered separately from those relating to MS, because of the complexity of the metabolic syndrome, which includes several alterations of metabolic conditions not specifically associated with diabetes.

#### 3.1.1. *In Vivo* Methods

Table 1 reports the *in vivo* methods used in clinical trials. The preferred type of clinical study to evaluate the anti-inflammatory effect of PFS in humans was the double-blind randomized controlled clinical trial.

Several publications reported in the present review used randomization in clinical trials, but in a few cases, randomization was not described in detail or incompletely applied (e.g., no randomization for age or gender). Positive aspects of randomization include the elimination of biases, balanced arms, and the capacity to form the basis for statistical tests.

A method of randomization should be considered appropriate if it allows each study participant to have the same chance of receiving the intervention [22]. Methods of allocation using the date of birth or of admission, hospital numbers, or alternation are not appropriate. Suitable methods for randomization include using a table of random numbers or computer generation. In most studies, the subjects enrolled included people of both sexes. Studies of only male or female subjects do not reflect the whole population and the results are not reliable. Almost all studies reported the number of dropouts, but it was not always clear if this was due to lack of efficacy or due to adverse effects. The analytical method used to quantify inflammatory markers such as CRP, cytokines, malondialdehyde (MDA), and adhesion molecules was reported in most studies. In several cases, methods were well described, and manufacturers' protocols were appropriate. The methods most frequently applied were the ELISA test, immunoturbidimetry, and real-time PCR.

#### 3.1.2. Inflammatory Biomarkers Affected by PFS in Metabolic Syndrome

The inflammatory parameters decreasing after PFS treatment were also reviewed. Although changes after PFS treatment depend on the plant used and on the bioavailability of the active compounds and the method conditions, parameters that change in a short period of time can be selected as useful biomarkers of the anti-inflammatory properties of PFS. In patients affected by MS (Table 1), CRP levels decreased in three of the seven studies measuring it: a randomized open controlled cross-over study involving 482 patients for eight weeks [18], a 10-week randomized double-blind controlled clinical trial involving 60 patients [16], and a 1-year cross-sectional study involving 486 patients [21].

On the contrary, the other four of these studies [17, 19, 20, 23] reported no changes in CRP values. This discrepancy could be due to the nature of the botanicals used, the bioavailability of active compounds, the number of patients enrolled in the study, the type of study, and/or the laboratory methods for biomarker measurement. A larger number of studies is necessary to assess if CRP is a suitable parameter to evaluate the decrease of inflammatory status in MS after treatment with PFS. 

E-selectin levels were evaluated in four studies [17–19, 23], but a decrease was recorded in only one [18], which rules out this biomarker as suitable for the purpose. TNF-*α* levels were evaluated in three studies [16, 18, 19], and two of them [16, 18] reported a significant reduction after treatment with soy proteins (8 weeks) and fresh algae infusion (10 weeks). No significant differences before and after treatment with berry derivatives were found [19]. IL-18 was evaluated in only one study [18], and no conclusions can be drawn. IL-6 levels: two studies [17, 23] reported no significant differences after 5 and 9 weeks; another three studies [16, 18, 20] reported a significant reduction in both gene expression and serum after 8, 10, and 20 weeks. 

We may conclude that TNF-*α* and IL-6 may be suitable biomarkers to evaluate in a rather short time (6–10 weeks) the improvement of the inflammatory status in humans after PFS treatment, but careful consideration of the botanical formulation used in the study (i.e., kind of extract, occurrence of active principles, and their bioavailability) and the amount of PFS taken before should be given before ruling a proinflammatory biomarker in or out. Additional studies are needed before considering CRP and IL-18 as biomarkers modulated by PFS treatment, since the data occurring in the literature do not allow us to draw clear conclusions. The importance of IL-6, TNF-*α*, and CRP as proinflammatory biomarkers is well documented. TNF-*α* is released by adipose tissue and is overexpressed in obesity, and TNF-*α* can modulate insulin resistance in a variety of clinical trials related to obesity [6, 15, 24]. CRP and IL-6 are peripheral inflammatory markers, and their measurement improves the prediction of the risk of cardiovascular events [24].

### 3.2. Diabetes Studies

#### 3.2.1. *In Vitro* Methods

Table 2 reports the *in vitro* methods applied to investigate inflammatory markers in diabetes. The method mostly used for the assessment of beneficial effects of botanicals/plant food supplements on diabetes were based on quantitative PCR (qPCR), a real-time PCR coupled with reverse transcriptase. This is a very sensitive and specific method for analyzing mRNA levels as a marker of gene expression. Uemura et al. [25] reported the use of SYBR Green assay, while Chuang et al. [26, 27] and Cao et al. [28] reported the use of the highly specific and sensitiveTaQman assay. Other frequently used methods are ELISA assay, immunoblotting, and transfections, in a variety of cell cultures, with a plasmid containing the luciferase reporter gene under the control of the NF-*κ*B responsive element(pNF-*κ*B-luc);the latter method is widely used for *in vitro* assays because it assesses events upstream of the inflammatory cascade which activates the NF-*κ*B pathway.

In the luciferase transfection assay only, Chuang et al. [26, 27] used primary cultures (from humans or animals) in their experiments; they are considered the most predictive, as primary cells retain the characteristics of the starting tissue. However, the isolation of appropriate cells from primary cultures can be difficult as the cell population is heterogeneous. Moreover, primary cultures have a limited life. Considering the challenges associated with modelling a chronic disease such as diabetes, it is encouraging to see that several *in vitro* methods for investigating inflammation have been developed, but few of them have been applied to the evaluation of PFS benefit assessment. In this sense, it would be interesting to develop methods to evaluate the following inflammatory events:

1. nuclear translocation of NF-*κ*B factor in the nucleus (usually evaluated by ELISA test) and the consequent transcription of inflammatory genes and the expression of adhesion molecules on endothelium, leading to the vascular complications of diabetes;
2. expression of adhesion molecules (i.e., sICAM-1, sICAM-2 and sVCAM-1, and E-selectin), which are typically overexpressed in diabetes and in cardiovascular disease [38]. Adhesion molecule expression is usually measured by RT-PCR (mRNA levels) and ELISA (protein expression on the cell surface);
3. evaluation of metalloproteinase-9 (MMP-9) secretion and gene expression. MMP-9 is induced by hyperglycemia and accelerates some diabetic complications such as retinopathy [39]. MMP-9 gene expression is usually measured by RT-PCR, secretion and enzymatic activities by zymography or western blotting;
4. evaluation of monocyte-macrophage chemotaxis in the endothelium. Indeed, monocytes and macrophages play a role in accelerating diabetes and in the development of atherosclerosis. They express specific receptors for advanced glycation end products (AGEs), which are proteins or lipids that become nonenzymatically glycated and oxidized after contact with aldose sugars.

After the binding of AGEs and intracellular processing, monocytes/macrophages synthesize and secrete growth-promoting cytokines such as TNF-*α*, interleukin 1, and insulin-like growth factor, responsible for vascular complications in diabetes [40]. Furthermore, circulating AGEs may interact with endothelial receptors, which leads to perturbation of cellular properties, such as upregulation of the transcription and the translocation of nuclear factor NF-*κ*B [41]. Chemotaxis is generally measured under agarose [42, 43] or by chemotaxis assay, based on evaluation of cell migration through apposite filters after inflammatory stimuli have been placed in chambers (Boyden Chamber Assay) [44].

#### 3.2.2. *In Vivo* Methods

Table 3 reports the *in vivo* methods developed for human trials.

The majority of clinical trials evaluating the effect of botanicals on inflammatory biomarkers in diabetic patients were randomized, double-blind randomized controlled trials and randomized, open and controlled studies. The lack of blinding is particularly critical when the treatment is applied versus placebo. In fact, the placebo effect is a major cause of bias due to patient or doctor awareness [22]. Several studies have indicated that nonrandomized trials are more likely to yield a positive result for a new treatment than for an established conventional one. In some of the clinical trials considered, randomization was not described in detail or was unsuitable. In two papers [53, 54], the number of subjects was limited. In some studies [49, 51, 55], the dropout rate and the reasons for it were not critically discussed. The most often used methods for biomarker quantification were ELISA assay and HPLC analysis.

#### 3.2.3. Inflammatory Biomarkers Affected by PFS in Diabetes

The parameters most frequently measured in clinical trials were as follows: CRP = MDA > endothelin-1 = urinary thromboxane B_2_ metabolites > IL-6, IL-18, TNF-*α*, and PGI_2_, 8-isoprostane. Six studies [31, 47–50, 55] examined CRP levels; among them, three [31, 47, 50] reported a significant reduction in serum CRP levels after 4 weeks of treatment, two studies reported no significant changes [48, 49], and one reported increased levels of CRP [55]. As for MS, further studies are mandatory before conclusions on the efficacy of *in vivo* CRP measurement in diabetes after PFS treatment can be drawn.

Serum levels of malondialdehyde (MDA) were measured in three studies [46, 50, 56], and all reported a significant reduction after 4, 8, and 12 weeks of PFS consumption. MDA is an important biomarker of oxidative stress in diabetes as a consequence of persistent hyperglycemia and lipid peroxidation. These trials establish serum MDA measurement as appropriate for the purpose.

IL-6 levels were measured in three studies [48, 49, 56] and one reported a significant reduction after PFS treatment [56]. IL-18 [48] and TNF-*α* [56] each were measured in only one study, and in both cases, a significant decrease was found. Nevertheless, further studies are needed to evaluate IL-6 and IL-18 as suitable inflammatory parameters. Endothelin-1, generally overexpressed in diabetes models [57], was found to decrease in two studies [45, 56], after 12 and 8 weeks of treatment, respectively.

Two studies showed a significant reduction of thromboxane B_2_ (TXB_2_) urinary metabolites after 4 and 12 weeks of treatment, respectively [51, 54]. PGI_2_ [51] and 8-isoprostane [48] were each evaluated only in one study; both decreased after PFS treatment.TXB_2_,PGI_2_, and 8-isoprostane are key proinflammatory biomarkers in diabetes. They are massively released in the wake of hypercoagulation and the vascular modifications that are typical for this disease. The paucity of studies in which these mediators were assayed in response to PFS treatment does not allow us to establish their usefulness in investigating the benefit of PFS treatment.

## 4. Conclusions

The aim of the present paper was to collect and critically discuss the existing experimental approaches used *in vitro, in silico*, and* in vivo* for benefit assessment of botanicals or plant food supplements (PFSs) in decreasing inflammation in MS-related diseases. PFS often consist of a complex mixture of compounds, which makes benefit assessment difficult and subject to interferences and false positives. The development of reliable methods for evaluating benefit assessment and their application and validation is therefore crucial.

No *in silico* methods were found in MS-related diseases. qRT-PCR was the *in vitro* method most widely applied for measuring the expression of inflammatory cytokines in diabetes, but no *in vitro* methods were found for testing PFS benefits in MS. Data from the *in vivo* studies (clinical trials) show that the inflammatory markers CRP, MDA, and PGI_2_ are likely to be useful in judging the efficacy of PFS treatment, although more studies are needed to validate this conclusion. In addition, two proinflammatory biomarkers, IL-6 and TNF-*α*, were the only two parameters to change in both *in vitro* and *in vivo *systems, and these biomarkers should be carefully considered in future studies.

## Figures and Tables

**Table 1:** Inflammatory parameters measured in metabolic syndrome.

Method^1^	Botanical or botanical derivatives used	No. of participants and length of treatment	Parameters measured	Results	Reference
Double-blind randomized controlled cross-over trial	Olive oil (*Olea europaea* L.)	20 (6-week washout period plus 4-h study session)	Expression of inflammatory genes (CCL3, CXCL1, CXCL3, CXCR4, IL-1*β*, IL-6, and OSM	Expression of inflammatory genes was reduced	Camargo et al., 2010 [15]
Randomized double-blind controlled trials	ProAlgaZyme (freshwater algae infusion)	60 (10 weeks)	hs-CRP, IL-6, and TNF-*α*	Significant reduction of all parameters	Oben et al., 2007 [16]
Single-blind randomized controlled trial	Plant sterol margarines (30 g/day)	53 (5 weeks)	CRP, IL-6, CD 40 ligand (CD40L), and E-selectin	No changes	Gagliardi et al., 2010 [17]
Randomized open controlled cross-over trials	Soy nut/soy proteins	482 (8 weeks)	Serum endothelin-1, sICAM-1, sVCAM-1, E-selectin, IL-2, IL-6, IL-18, TNF-*α*, SAA, and CRP	Reduction of CRP for soy protein, E-selectin, TNF-*α*, IL-18, and CRP for soy nuts	Azadbakht et al., 2007 [18]
Randomized open controlled clinical trials	Berries and derivatives*	61 (20 weeks)	TNF-*α*, ICAM, VCAM, E-selectin, and hs-CRP	No significant differences	Lehtonen et al., 2010 [19]
Open nonrandomized noncontrolled clinical trials	Freeze-dried strawberry extract	35 (4 weeks)	Malondialdehyde (MDA), hs-CRP, and adiponectin	No significant differences	Basu et al., 2009 [20]
Cross-sectional studies	Fruit food group** Vegetable fruit group***	486 (1 year)	CRP	Reduction of CRP for both groups	Esmaillzadeh et al., 2006 [21]

*Participants consumed lingonberry, bilberry, black currant, sea buckthorn as such or as derivatives (juice, oil, and powder).

**Pears, apricots, cherries, apples, raisins or grapes, bananas, cantaloupe, watermelon, oranges, grapefruit, kiwi, strawberries, peaches, nectarines, tangerines, mulberry, plums, persimmons, pomegranates, lemons, pineapples, fresh figs, and date.

***Vegetable fruit group: cabbage, cauliflower, Brussels sprouts, kale, carrots, tomatoes, spinach, lettuce, cucumber, mixed vegetables, eggplant, celery, green peas, green beans, green pepper, turnip, corn, squash, mushrooms, and onions.

^1^Clinical studies used to assess inflammatory parameters are listed from the best to the worst method applied.

**Table 2:** *In vitro* methods and inflammatory parameters measured in diabetes.

Method	Botanical used	Parameters measured	Results	Reference
Flow cytometry	Peanut oil (*Arachis hypogaea*)	Apoptosis induced by TNF-*α* in rat *β*-pancreatic cell line (INS-I)	Reduction of apoptosis	Vassiliou et al., 2009 [29]
Luciferase assay	*Sinocrassula indica* Berger (Shilianhua) extract	Transcriptional activity of NF-kB induced by LPS in RAW264.7 macrophages	Reduction of transcriptional activity of NF-kB	Yin et al., 2009 [30]
Quantitative polymerase chain reaction (qRT-PCR)	*Zizyphus lotus* L. Desf. extract	mRNA levels of IL-2 in human (Jurkat) T cells stimulated with anti-CD3 antibodies	Reduction of mRNA and IL-2 levels	Uemura et al., 2010 [25]
	Lyophilized grape powder (*V. vinifera*)	Gene expression of IL-6, IL-8, IL-1*β*, MCP-1, COX-2, and TLR-2 in primary human adipocytes stimulated with TNF-*α*	Attenuation of all parameters measured	Chuang et al., 2011 [26]
	White grape seed extract (*V. vinifer*a)	Gene expression of CYP, PPAR*γ*, LEP, APM1, IL-6, and MCP-1 in THP-1 cells and human adipocytes stimulated with LPS and TNF-*α*, respectively	Reduction of IL-6 and MCP-1 expressions; modulation of APM1 and LEP (adipokine) gene expressions	Kar et al., 2009 [31]
	Lyophilized grape powder (*V. vinifera*)	Gene expression of IL-6, IL-8, IL-1*β*, and TNF-*α* in human macrophages and adipocytes stimulated with LPS	Reduction of IL-6, IL-8, IL-1*β*, and TNF-*α* gene expressions	Chuang et al., 2010 [27]
	*Cinnamomum burmannii* extract	Expression of tristetraprolin (TTP) in mouse RAW264.7 macrophages treated with LPS	Induction of TTP	Cao et al., 2008 [28]
	*Crataegus pinnatifida* Bunge var. *typica* Schneider and *C. pinnatifida *Bunge	Gene expression of iNOS and COX-2 in murine RAW264.7 macrophages treated with LPS	mRNA levels of iNOS and COX2 were inhibited by *C. pinnatifida* Bunge	Li et al., 2010 [32]
	Fructus Xanthii	Protection from IL-1*β* and IFN-*γ*and NF-kB induced in pancreatic cell line RINM5F	Complete protection	Song et al., 2009 [33]
	Lyophilized grape powder (*V. vinifera*) obtained from red, green, and blue-purple seeded and seedless California table grapes	Concentration of IFN-*γ* inducible protein-10 (IP-10) in human adipocytes and macrophages after treatment with LPS	Reduction of IP-10	Chuang et al., 2010 [27]
	Palmitic acid, oleic acid, or DHA	Production of TNF-*α* and IL-10 in 3T3-L1 murine adipocytes	Increase of IL-10, no effect on TNF-*α*	Bradley et al., 2008 [34]
	Lyophilized grape powder, (*V. vinifera*)	Activation of NF-kB factor and Ik*α* degradation in primary cultures of human adipocytes treated with TNF-*α*	Reduction of NF-kB activity and mediation of Ik*α* degradation	Chuang et al., 2011 [26]
	Lyophilized grape powder, (*V. vinifera*)	Activation of NF-*κ*B factor in primary cultures of human adipocytes and macrophages treated with LPS	Inhibition of NF-kB activation	Chuang et al., 2010 [27]
	*Cinnamomum burmanii *	Cytokine (TNF-*α*, IL-6, and COX-2) production by mouse RAW 264.7 macrophages treated with LPS	Reduction of cytokine (TNF-*α*, IL-6, and COX-2) production	Cao et al., 2008 [28]
Chromogenic assay (TMPD)	Extract of rhizome of *Zingiber officinale* Roscoe	Enzymatic activity of COX in C2C12 cells	Reduction of enzymatic activity	Priya Rani et al., 2011 [35]
ELISA	Diosgenin from seeds of *Trigonella foenum-graecum *	Levels of adiponectin and MCP-1 in 3T3-L1 preadipocytes	Increase of adiponectin levels and decrease of MCP-1	Uemura et al., 2010 [25]
Caspase-3 activity luminometric assay	*Hypericum perforatum* L.	Determination of apoptosis in rat insulinoma cell line (INS-1E) stimulated with cytokines	Inhibition of apoptosis	Menegazzi et al., 2008 [36]
	White grape seed extract (*V. vinifera*)	P65 translocation and PIKB*α* protein in human monocyte cell line THP-1 and human adipocyte treated with LPS and TNF-*α*	Partial inhibition	Chac$\overset{´}{\text{o}}$n et al., 2009 [37]
No production colorimetric assay	*Crataegus pinnatifida* Bunge var. *typica* Schneider and *C. pinnatifida* Bunge	Cytotoxicity and no inhibitory activity in murine RAW 264.7 macrophages stimulated with LPS	Inhibition of cytotoxicity and no activity only by *C. pinnatifida* Bunge	Li et al., 2010 [32]
Nitrite measurement colorimetric assay	Fructus Xanthii	No production in RINm5F cells treated with IL-1*β* and IFN-*γ*	Inhibition of no production	Song et al., 2009 [33]
NF-kB binding assay	Dietary fatty acids	Binding activity of the P65 subunit of NF-kB in nuclear extracts from 3T3-L1 murine adipocytes	Decrease of binding activity	Bradley et al., 2008 [34]

**Table 3:** Inflammatory parameters assessed in clinical trials of botanicals in diabetes.

Method^1^	Botanical or botanical derivatives used	No. of participants and length of treatment	Parameters measured	Results	Reference
Double-blind randomized controlled cross-over trial	Grape seed extract (*V.vinifera*)	32 (4 weeks)	hs-CRP	Decreased hs-CRP	Kar et al., 2009 [31]
Double-blind randomized controlled multicenter trial	Pycnogenol (extract of bark from the French Maritime Pine, *Pinus pinaster*)	77 (12 weeks)	Endothelin-1 and ketoprostaglandin F1-*α*	Decreased endothelin-1 and increased ketoprostaglandin F1-*α*	Liu et al., 2004 [45]
Randomized double-blind controlled trial	Pomegranate (*Punica granatum* L.), green tea (*C. sinensis* L.) extract	114 (12 weeks)	Plasma MDA	Decrease of MDA	Kutan Fenercioglu et al., 2010 [46]
	Blueberry (*Vaccinium arctostaphylos* L.) leaves water extract	42 (4 weeks)	Serum CRP	Decreased hs- CRP	Abidov et al., 2006 [47]
Single-blind randomized controlled trial	Coffee	47 (1-month washout, 1 month 4 cups/day, and 1 month 8 cups/day)	CRP, IL-6, IL-1, IL-18, 8-isoprostane, MIF, adiponectin, leptin, and SSA	Decreased IL-18, 8-isoprostane and increased adiponectin and other markers unchanged	Kempf et al., 2010 [48]
Randomized controlled cross-over trial	Green tea (*C. sinensis* L.) aqueous extract	55 (4 weeks)	hs-CRP and major cytokine mediator (IL-6)	Both mediators unchanged	Ryu et al., 2006 [49]
Randomized open controlled clinical trial	Black tea (*C. sinensis*)	46 (4 weeks: 150 mL week 1, 300 mL week 2, 450 mL week 3, and 600 mL week 4)	Serum CRP, MDA, and fibrinogen	Decreased serum CRP after consumption of 600 mL and MDA after consumption of 300 mL; fibrinogen unchanged	Neyestani et al., 2010 [50]
Open noncontrolled nonrandomized clinical trial	*Ginkgo biloba* extract (24% ginkgo flavone glycosides and 6% terpenes	47 (12 weeks)	Urinary metabolites of thromboxane B2 (TXB_2_)and prostacyclin (PGI2)	Decreased urinary TXB_2_and PGI2	Kudolo et al., 2003 [51]
Prospective cohort studies	Coffee	2040 (6 years)	Adiponectin	Higher in drinkers of >4 cups/day	Williams et al., 2008 [52]

Macrophage migration inhibitory factor (MIF) and malondialdehyde (MDA).

^1^Clinical studies used to assess inflammatory parameters are listed from the best to the worst method applied.
